# IMPLEMENTATION BOOSTER: A FRONTLINE INNOVATION USING AN AFHS-QUESTIONNAIRE

**DOI:** 10.1093/geroni/igae098.0479

**Published:** 2024-12-31

**Authors:** Heba Aldossary, Holly Kouts, Mary Dolansky

**Affiliations:** Case Western Reserve University, Cleveland, Ohio, United States; CVSHealth Minute Clinic, Woonsocket, Rhode Island, United States; Case Western Reserve University, Cleveland, Ohio, United States

## Abstract

Booster strategies originating from frontline providers are essential for optimizing 4Ms care delivery. The Age-Friendly Health Systems Questionnaire (AFHS-Q) is a novel approach, developed by frontline providers in the MinuteClinic, that enhances efficiency by having patients complete eight 4Ms-related questions while awaiting their medical visit. This approach fosters patient engagement, allowing individuals to express their concerns and preferences, ensuring personalized care. Providers (nurse practitioners and physician associates) across the ~1,100 MinuteClinic sites nationwide received AFHS-Q kits. Survey sent to 3,300 nurse practitioners completing the 4Ms and documenting findings before implementation. After four weeks, we surveyed 100 providers to provide feedback (scale range 1 to 5). 91 providers completed the survey. In general, feedback on the AFHS-Q was positive relating to the comfort of use (M = 4.18, SD =.99), usefulness (M = 3.97, SD = 1.10), acceptability (M = 4.12, SD =.83), and effort for use (M = 2.75, SD = 1.17). Providers confidence in completing the 4Ms (before AFHS-Q: M = 3.92, SD = 1.90; after AFHS-Q: M = 4.18, M =.97) and documenting the 4Ms before AFHS-Q: M = 3.84, SD =.99; after AFHS-Q: M = 4.17, M =.91) was significantly higher following AFHS-Q use. Providers also reported a significant reduction in time for visits after using the AFHS-Q. In summary, AFHS-Q proves effective, feasible, and well-received. Valuable feedback drives continuous refinement. Our findings emphasize the importance of provider-driven solutions for evidence-based healthcare quality improvement efforts.

